# Facial emotion identification impairments in Chinese persons living with schizophrenia: A meta-analysis

**DOI:** 10.3389/fpsyt.2022.1097350

**Published:** 2022-12-20

**Authors:** Yan-Min Xu, Fang Deng, Bao-Liang Zhong

**Affiliations:** ^1^Department of Psychiatry, Wuhan Mental Health Center, Wuhan, China; ^2^Department of Clinical Psychology, Wuhan Hospital for Psychotherapy, Wuhan, China

**Keywords:** facial emotion identification, schizophrenia, case-control studies, Chinese, meta-analysis

## Abstract

**Background:**

Facial emotion identification (FEI) deficits are associated with impaired social functioning in persons living with schizophrenia (PLwS), but the research on emotion-specific FEI deficits remains inconclusive. Furthermore, existing studies on FEI deficits are limited by their small sample sizes. We performed a meta-analysis of studies comparing the FEI abilities between Chinese PLwS and healthy controls in terms of the six basic facial emotions (happiness, sadness, fear, disgust, anger, and surprise), as well as contempt, calmness, and neutral facial expressions.

**Methods:**

Major Chinese- and English-language databases were searched to retrieve case-control studies that compared the FEI task performance between Chinese PLwS and healthy controls (HCs) and reported the emotion-specific correct identification scores for PLwS and HCs. The Joanna Briggs Institute Critical Appraisal Checklist for Case-control Studies (“JBI checklist,” hereafter) was used to assess the risk of bias (RoB) of the included studies. Statistical analysis was performed using the “meta” package of R 4.1.2.

**Results:**

Twenty-three studies with a total of 28 case-control cohorts and 1,894 PLwS and 1,267 HCs were included. The RoB scores of the included studies ranged from two to seven. PLwS had statistically significantly lower FEI scores than HCs and the corresponding emotion-specific pooled standard mean differences (95% confidence intervals) were −0.69 (−0.88, −0.50) for happiness, −0.88 (−1.12, −0.63) for sadness, −1.44 (−1.83, −1.06) for fear, −1.18 (−1.60, −0.76) for disgust, −0.91 (−1.24, −0.57) for anger, −1.09 (−1.39, −0.78) for surprise, −0.26 (−0.51, −0.01) for contempt, −0.31 (−0.52, −0.09) for calmness, and −0.42 (−0.65, −0.18) for neutral. In the analyses of sources of heterogeneity, drug-naïve status, clinical setting, positive and negative psychotic symptoms, and RoB were significant moderators of the magnitudes of FEI deficits.

**Conclusions:**

Chinese PLwS have significant FEI impairments in terms of recognizing the six basic facial emotions, contempt, calmness, and neutral emotions, and the magnitude of impairment varies depending on the type of emotion, clinical characteristics, and the level of RoB of the study. It is necessary to consider the characteristics of FEI deficits and the clinical moderators in the FEI deficits to develop remediation strategies targeting FEI deficits in schizophrenia.

## Introduction

Facial emotion recognition (FER) impairments are a rather stable trait of schizophrenia, which has been associated with impaired social functioning and predicts subsequent declines in work functioning, social participation, and abilities of independent living in persons living with schizophrenia (PLwS) (1–5). Nevertheless, there has been accumulating evidence that certain psychological and cognitive training interventions are effective for mitigating FER impairments and further result in large improvements in social functioning in PLwS (6–12). Therefore, FER ability has been a promising treatment goal for effective psychosocial rehabilitation in schizophrenia. To optimize the development and selection of remediation strategies targeting FER deficits in schizophrenia, it is necessary to have adequate knowledge of the characteristics of FER difficulties in PLwS.

In the literature, FER deficits in schizophrenia have been extensively examined; however, controversy still exists regarding the specificity of FER deficits (i. e., specific to FER only or in both FER and non-emotional face processing) and the moderating roles of clinical factors on FER deficits (i.e., whether paranoid and non-paranoid schizophrenia differ in FER deficits) (13–17). Two published systematic reviews and meta-analyses pooled effect sizes of the differences in overall FER abilities between PLwS and healthy controls and demonstrated the general deficit in both facial emotion perception and processing in schizophrenia and the significant study-level associations of FER deficits with negative psychotic symptoms, inpatient hospitalization, and late age at onset of schizophrenia (14, 16). Nonetheless, the two systematic reviews focused on the total FER only and directly pooled the effect sizes from different FER tasks together, which ignored the heterogeneity across tasks [i.e., facial emotion identification (FEI) and discrimination], so their meta-analytic findings were still not detailed enough. Since prior studies report conflicting findings on FER deficits in a specific emotion (i.e., happiness) and across a variety of FER tasks (13, 18–20), the specificity of FER deficits with respect to the category of emotion and type of FER task remains inconclusive. For example, two published studies have consistent findings on the significantly lower correct disgust and fear FEI rates in Chinese PLwS than healthy controls but have inconsistent findings on the FEI of happiness: one found comparable rates between Chinese persons with first-onset schizophrenia and healthy controls, and the other found significantly lower rates in Chinese PLwS than healthy controls (18, 21). Importantly, the unstable findings may also be ascribed to the small sample sizes and the inadequate statistical powers of prior studies.

To further clarify the specificity of FER impairments and advance our understanding of the mechanisms of FER impairments in schizophrenia, we performed a meta-analysis of case-control studies using the FEI task to assess the FER deficits in terms of six basic facial emotions (happiness, sadness, fear, disgust, anger, and surprise) and contempt, calmness, and neutral facial expressions in Chinese PLwS. Schizophrenia is typically characterized by language disturbances and semantic deficits and the completion of FEI tasks relies on language and semantic skills (22–25), so experimental FER paradigms of studies to be included were limited to FEI tasks only. To minimize the clinical heterogeneity in FEI deficits caused by race and culture (26–28), the included studies were limited to those with Chinese participants.

## Methods

This meta-analysis was reported according to the PRISMA guideline (29). Literature search, the inclusion of eligible studies, data extraction, and risk of bias (RoB) assessment were independently performed by the first and second authors of this study, and disagreements were addressed *via* discussion and consensus with the corresponding author.

### Inclusion and exclusion criteria

Case-control studies that compared the FEI task performance between Chinese PLwS and healthy controls and reported correct identification scores (rates or crude scores, mean ± standard deviations [SDs]) in terms of any of the above-mentioned nine emotions were considered eligible for this study. Studies that did not include healthy controls, used facial emotion discrimination tasks only, examined FER abilities under different conditions, employed eye emotion recognition tasks, adopted prosodic emotion recognition tasks, or did not provide meta-analyzable data were excluded.

### Literature search

A literature search was conducted within both Chinese- and English-language databases from their inception to November 13, 2022: CNKI, Wanfang, VIP Information, PubMed, Embase, and PsycINFO. The main search terms were as follows: (“facial emotion” OR “facial affect” OR “emotional face” OR “emotional expression” OR “facial expression”) AND “schizophreni^*^” AND (“identification” OR “recognition” OR “perception”) AND (“Chin^*^” OR “Taiwan” OR “Hong Kong”). Reference lists of included studies and related reviews were also manually searched to avoid missing studies.

### Data extraction

A standardized form specifically developed for this study was used to extract data from included studies. Extracted variables included first author, publication year, diagnostic criteria of schizophrenia, numbers of participants in the case and control groups, clinical characteristics of the case group (i.e., mean age, proportion of men, and clinical stage of schizophrenia), characteristics of the FEI task (i.e., facial emotion database and classification of facial emotion), indicators of RoB assessment (i.e., the validity of the FEI task), and emotion-specific correct identification scores of the FEI task (means ± SDs).

### RoB assessment

The Joanna Briggs Institute Critical Appraisal Checklist for Case-control Studies (“JBI checklist,” hereafter) was used to assess the RoB of the included studies (30). The JBI has 10 methodology items of a case-control study: comparability, matching, identification of cases and controls, validity of exposure measure, method of exposure measurement, identification of confounders, handling of confounders, validity of outcome measurement, exposure period, and statistical analysis. These items were assessed on four-choice options (yes, no, unclear, and not applicable), and one point was assigned to a “yes” response. Since the item “exposure period” was not applicable and removed from the RoB assessment, the total RoB score in our study ranged from zero to nine, with higher scores suggesting lower RoB.

### Statistical analysis

Meta-analysis was used to synthesize standardized mean differences (SMDs) and their 95% confidence intervals (CIs) for the magnitudes of the differences in correct identification scores between schizophrenia patients and healthy controls because the identification abilities were expressed in two distinct ways in included studies: correct rates in some studies and crude correct scores in other studies. Forest plots were generated to show SMDs and the combined estimates. When there was evidence of heterogeneity (*I*^2^ > 50% or *P* < 0.10 for *Q* statistics), the random-effect model was adopted to combine SMDs; otherwise, the fixed-effect model was used to combine SMDs. In the present study, the SMD was equivalent to the effect size measure, Hedges' *g*, with absolute values of 0.20–0.49, 0.50–0.79, and 0.80+ denoting small, medium, and large differences, respectively (31–33).

Sources of heterogeneity in the pooled SMDs were examined by using subgroup analyses according to potential categorical moderators (diagnostic criteria, clinical stage of schizophrenia, status of antipsychotic treatment, clinical setting, task, and type of correct identification score) and univariate meta-regression analyses according to potential continuous moderators [publication year, mean age of the schizophrenia sample, mean years of education of the schizophrenia sample, % of males in the schizophrenia sample, mean Positive and Negative Syndrome Scale positive symptom subscale (PANSS-P) score of the schizophrenia sample, mean PANSS negative symptom subscale [PANSS-N] score, and RoB score]. In studies assessing psychotic symptoms by using the Scale for the Assessment of Positive Symptoms (SAPS) and the Scale for the Assessment of Negative Symptoms (SANS), the recommended conversion equations were used to convert SAPS and SANS scores into PANSS-P and PANSS-N scores, respectively (34). Funnel plots and Egger's and Begg's tests were used to test publication bias. Two-sided *P* < 0.05 was considered statistically significant. All analyses were conducted by using R 4.1.2 (R Development Core Team; Vienna, Austria).

## Results

**Figure 1:**
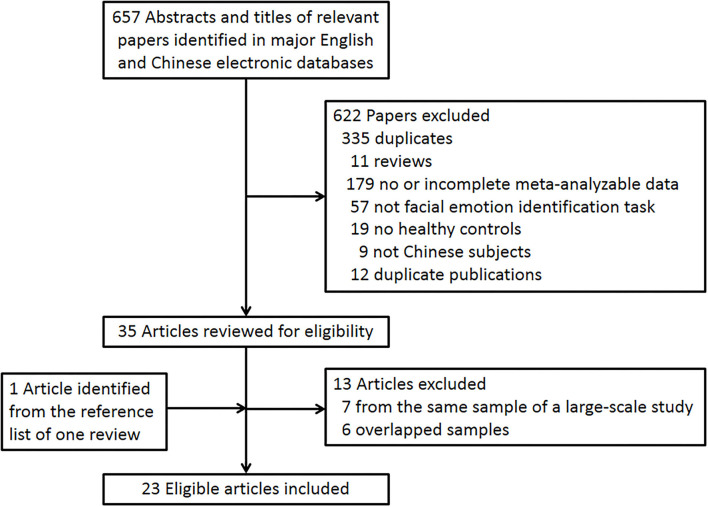
Flowchart of study inclusion.

The literature search initially identified 657 records, and finally, 23 studies with a total of 28 case-control cohorts were included (21, 35–56) (Figure 1). There were 1,894 PLwS and 1,267 healthy controls in the included studies. The RoB scores of the included studies ranged between two and seven, with a median score of four. Detailed characteristics and RoB scores of the 23 included studies are shown in Table 1.

**Table 1:** Characteristics and risk of bias scores of included studies.

**References**	**Participants (*n*, mean age, male/female)**	**Diagnostic criteria**	**Clinical characteristics**	**Clinical setting**	**Facial emotion identification task**	**Emotion categories**	**Identification measure**	**Risk of bias score**
Dong et al. (35)	SCH (65, 28 years, 17/48); HC (67, NR, 20/47)	CCMD-3	SCH	Outpatient & inpatient	Chinese facial emotion test	Happiness, sadness, fear, disgust, anger, surprise	Correct rate	4
Chen et al. (36)	SCH (42, 29.7 years, 42/0); HC (37, 32.0 years, 37/0)	CCMD-3	SCH	Inpatient	Chinese facial expression video system	Happiness, sadness, neutral	Correct rate	4
Dong et al. (37)	SCH (121, 28.3 years, 35/86); HC (76, 30.2 years, 26/50)	CCMD-3	Acute SCH	Outpatient & inpatient	Chinese facial emotion test	Happiness, sadness, fear, disgust, anger, surprise	Correct score	5
Gao et al. (38)	SCH (61, 27.4 years, 17/44); HC (57, 28.9 years, 21/36)	CCMD-3	SCH	Outpatient & inpatient	Chinese facial emotion test	Happiness, sadness, fear, disgust, anger, surprise	Correct score	4
Dong et al. (39)	SCH (82, 28.3 years, 22/60); HC (88, 29.3 years, 27/61)	CCMD-3	Drug-naïve acute SCH	Outpatient & inpatient	Chinese facial emotion test	Happiness, sadness, fear, disgust, anger, surprise	Correct score	5
Zhang and Chen (40)	SCH (100, 35.7 years, 55/45); HC (100, 34.3 years, 60/40)	CCMD-3	Remitted SCH	Outpatient & inpatient	Chinese facial emotion test	Happiness, sadness, fear, disgust, anger, surprise	Correct score	5
Tse et al. (41)	SCH (40, 40 years, 20/20); HC (46, 39 years, NR)	DSM-IV	Remitted SCH	Outpatient	Facial affect perception task	Happiness, sadness, anger, neutral	Correct score	6
Leung et al. (21)	First-onset SCH (50, 20.7 years, 25/25); HC (26, 21.7 years, 12/14) Chronic SCH (51, 43.5 years, 31/20); HC (28, 44.8 years, 17/11)	DSM-IV	Stable first-onset SCH Stable chronic SCH	Outpatient	Japanese and Caucasian facial expressions of emotion	Happiness, sadness, fear, disgust, anger, surprise	Correct rate	6
Yu (42)	SCH (88, 23.3 years, 50/38); HC (75, 23.2 years, 33/42)	ICD-10	Acute paranoid SCH	Inpatient	Japanese and Caucasian facial expressions of emotion	Happiness, sadness, fear, disgust, anger, surprise	Correct rate	2
Li (43)	SCH (25, 15.2 years, 19/6); HC (25, 15.3 years, 19/6)	DSM-V	Drug-naive type II SCH	Outpatient & inpatient	Basic facial expression cognition test for Chinese	Happiness, sadness, fear, disgust, anger, surprise, neutral	Correct score	4
Song et al. (44)	SCH (44, 35.5 years, 20/24); HC (41, 32.4 years, 17/24)	DSM-IV	Stable SCH	Inpatient	Computerized facial emotion recognition test	Happiness, sadness, fear, anger, contempt	Correct rate	7
Wang and Kang (45)	SCH (45, 32 years, 0/45); HC (45, 32 years, 0/45)	ICD-10	SCH	Inpatient	Ekman-Friesen pictures of facial affect	Happiness, anger, fear	Correct rate	4
Tang et al. (46)	Deficit SCH (37, 49.2 years, 37/0); non-deficit SCH (57, 46.5 years, 57/0); HC (54, 47.6 years, 54/0)	DSM-IV	Stable deficit SCH Stable non-deficit SCH	Inpatient	Chinese facial emotion test	Happiness, sadness, fear, disgust, anger, surprise	Correct score	6
Zhu et al. (47)	SCH (30, 33.5 years, 17/13); HC (30, 33.8 years, 15/15)	DSM-IV	Drug-naïve SCH	Inpatient	Chinese facial emotion database	Happiness, sadness, fear, disgust, anger, surprise	Correct rate	4
Lv (48)	SCH (31, 23 years, 20/11); HC (25, 21.4 years, 17/9)	DSM-IV	Drug-naïve first-onset SCH	Inpatient	Japanese female facial expression dataset	Happiness, fear, anger, neutral	Correct rate	2
Yang et al. (49)	SCH (30, 22.3 years, 15/15); HC (30, 24.6 years, 15/15)	DSM-IV	First-onset SCH	Inpatient	Ekman-Friesen pictures of facial affect	Happiness, fear, disgust	Correct rate	6
Zhu (50)	SCH (28, 34.7 years, 15/13); SCH (26, 34.7 years, 12/14); HC (30, 33.8 years, 16/14)	DSM-V	Drug-naive SCH	Inpatient	Chinese emotional face database	Happiness, sadness, fear, disgust, anger, surprise	Correct rate	4
Liu et al. (51)	Remitted SCH (65, 29.3 years, 35/30); Remitted SCH (45, 31.6 years, 26/19); HC (58, 31.4 years, 37/21)	CCMD-3	Remitted first-onset SCH Non-remitted first-onset SCH	Outpatient & inpatient	Chinese facial emotion test	Happiness, sadness, fear, disgust, anger, surprise	Correct score	4
Guo (52)	First-onset SCH (60, 27.6 years, 36/24); Chronic SCH (63, 30.2 years, 40/23); Chronic HC (50, 29.8 years, 27/23)	ICD-10	Drug-naive first-onset SCH Chronic SCH	Inpatient	Facial emotion recognition test	Happiness, fear, neutral	Correct rate	2
Zhao et al. (53)	SCH (162, 41.3 years, 74/88); HC (83, 39.7 years, 29/54)	DSM-IV	Stable SCH	Inpatient	Chinese facial emotion test	Happiness, sadness, fear, disgust, anger, surprise, neutral	Correct score	3
Du et al. (54)	SCH (60, 34.6 years, 18/42); HC (60, 37.3 years, 19/41)	DSM-IV	SCH	Inpatient	Chinese facial emotion images database with intensity classification	Happiness, sadness, fear, disgust, anger, surprise, neutral	Correct score	4
Gao (55)	SCH (35, 30 years, 14/21); HC (35, 29 years, 16/19)	ICD-10	Stable SCH	Outpatient & inpatient	Chinese affective picture system	Happiness, sadness, fear, anger	Correct rate	3
Lee et al. (56)	SCH (351, 45 years, 159/192); HC (101, 23.3 years, 37/64)	DSM-V	SCH	Outpatient & inpatient	Computerized adaptive test of facial emotion recognition	Happiness, sadness, fear, disgust, anger, surprise, neutral	Correct score	3

SCH, schizophrenia; HC, healthy controls; NR, not reported; CCMD-3, Chinese Classification of Mental Disorders, the third version; ICD-10, International Classification of Diseases, Tenth Revision; DSM-IV, Diagnostic and Statistical Manual of Mental Disorders, the fourth edition; DSM-V, Diagnostic and Statistical Manual of Mental Disorders, the fifth edition.

Results of the meta-analysis (Table 2) show that PLwS had statistically significantly lower FEI scores than healthy controls in terms of all the nine emotions of interest of this study and their corresponding SMDs (95%CIs) were −0.69 (−0.88, −0.50) for happiness, −0.88 (−1.12, −0.63) for sadness, −1.44 (−1.83, −1.06) for fear, −1.18 (−1.60, −0.76) for disgust, −0.91 (−1.24, −0.57) for anger, −1.09 (−1.39, −0.78) for surprise, −0.26 (−0.51, −0.01) for contempt, −0.31 (−0.52, −0.09) for calmness, and −0.42 (−0.65, −0.18) for neutral (Supplementary Figures 1–9).

**Table 2:** Results of meta-analysis on correct identification score differences between schizophrenia patients and healthy controls, as indicated by standardized mean differences (SMDs) and (95% confidence intervals, CIs).

**Emotion**	**Number of case-control cohorts**	**Number of participants (schizophrenia, healthy controls)**	**Heterogeneity (*I*^2^, *P*)**	**SMD (95% CI)**	**P**	**Publication bias**	
						**Egger's test (*****t***, ***P*****)**	**Begg's test (*****z***, ***P*****)**
Happiness^*^	28	1,894, 1,267	81.4%, <0.001	−0.69 (−0.88, −0.50)	<0.001	0.17, 0.866	−0.28, 0.779
Sadness^*^	22	1,665, 1,117	85.8%, <0.001	−0.88 (−1.12, −0.63)	<0.001	−0.56, 0.583	−0.82, 0.414
Fear^*^	26	1,812, 1,184	92.6%, <0.001	−1.44 (−1.83, −1.06)	<0.001	−1.14, 0.264	−0.37, 0.708
Disgust^*^	20	1,534, 988	87.8%, <0.001	−1.18 (−1.60, −0.76)	<0.001	−1.64, 0.119	0.00, 1.000
Anger^*^	24	1,699, 1,150	91.5%, <0.001	−0.91 (−1.24, −0.57)	<0.001	0.08, 0.939	−0.50, 0.620
Surprise^*^	19	1,504, 958	86.3%, <0.001	−1.09 (−1.39, −0.78)	<0.001	−1.95, 0.067	−1.50, 0.133
Contempt^**^	2	132, 116	0.0%, 1.000	−0.26 (−0.51, −0.01)	0.040	Not applicable	Not applicable
Calmness^**^	2	222, 143	0.0%, 1.000	−0.31 (−0.52, −0.09)	0.005	Not applicable	Not applicable
Neutral^*^	6	612, 284	50.0%, 0.075	−0.42 (−0.65, −0.18)	0.001	Not applicable	Not applicable

* Random-effects model.

** Fixed-effects model. Because the number of studies examining contempt, calmness, and neutral emotions are lower than 10, the publication bias of these studies was not tested.

Funnel plots of the six basic facial emotions were visually symmetrical (Supplementary Figures 10–15), and the results of both Egger's and Begg's tests suggested that there was no statistically significant publication bias across the included studies (*P* = 0.067–0.939, *P* = 0.133–1.000) (Table 2).

Diagnostic criteria, antipsychotic treatment status, clinical setting, and FEI task were identified as significant categorical moderators, while publication year, mean education years of the schizophrenia sample, % of men in the schizophrenia sample, mean PANSS-P score of the schizophrenia sample, mean PANSS-N score of the schizophrenia sample, and RoB score were identified as significant continuous moderators of the magnitudes of the FEI abilities between PLwS and healthy controls (Table 3). Specifically, the lowest significant pooled SMDs were shown in studies using DSM-V for happiness emotion, in studies using CCMD-3 for fear emotion, and in studies using DSM-IV for both disgust and surprise emotions, compared to studies using other diagnostic criteria from the same emotion-specific subgroups. Significantly lower pooled SMDs were observed in studies recruiting drug-naïve PLwS for the emotion of sadness, in studies enrolling both outpatients and inpatients with schizophrenia for both sadness and fear emotions, in studies recruiting inpatients with schizophrenia for anger emotion, and in studies adopting validated identification tasks in China in comparison to their counterparts from the same subgroups. There were significant positive correlations between happiness-specific pooled SMDs and % of men in the patient sample and fear-specific pooled SMDs and publication year while there were significant negative correlations between mean PANSS-P score in the patient sample and happiness-specific pooled SMDs, between mean PANSS-N score in the patient sample and sadness-specific pooled SMDs, between mean education years in the patient sample and fear-specific pooled SMDs, and between mean PANSS-N score in the patient sample and anger-specific pooled SMDs. RoB scores were significantly and negatively correlated with disgust-specific and surprise-specific pooled SMDs.

**Table 3:** Subgroup analyses and univariate meta-regression analyses of sources of heterogeneity in correct identification scores between schizophrenia patients and healthy controls, as indicated by standardized mean differences (95% confidence intervals) and coefficients (95% confidence intervals), respectively.

		**Emotion**					
		**Happiness**	**Sadness**	**Fear**	**Disgust**	**Anger**	**Surprise**
**Categorical moderator**							
Diagnostic criteria	CCMD-3	−0.73 (−1.11, −0.35)	−1.02 (−1.48, −0.55)	−1.85 (−2.43, −1.28)	−1.06 (−1.31, −0.82)	−0.79 (−1.00, −0.58)	−0.92 (−1.15, −0.69)
	DSM-IV	−0.45 (−0.72, −0.17)	−0.68 (−0.99, −0.36)	−1.63 (−2.48, −0.78)	−1.65 (−2.77, −0.53)	−1.07 (−1.65, −0.49)	−1.57 (−2.29, −0.86)
	ICD-10	−0.93 (−1.37, −0.49)	−1.24 (−2.79, −0.32)	−1.09 (−1.74, −0.45)	−0.29 (−0.60, 0.02)	−1.19 (−2.36, −0.03)	−0.16 (−0.47, 0.15)
	DSM-V	−0.94 (−1.15, −0.73)^*^	−0.85 (−1.06, −0.65)	−0.82 (−1.29, −0.34)^*^	−0.95 (−1.24, −0.66)^*^	−0.53 (−1.81, −0.75)	−0.85 (−1.04, −0.65)^*^
Stage of schizophrenia	Schizophrenia	−0.79 (−1.11, −0.47)	−0.82 (−1.17, −0.47)	−1.30 (−1.74, −0.85)	−1.09 (−1.24, −0.95)	−0.92 (−1.56, −0.28)	−1.12 (−1.42, −0.83)
	Acute schizophrenia	−0.82 (−1.34, −0.31)	−1.03 (−1.59, −0.47)	−1.70 (−2.92, −0.48)	−0.94 (−1.60, −0.29)	−0.63 (−1.11, −0.15)	−0.80 (−1.43, −0.17)
	Remitted schizophrenia	−0.56 (−1.15, 0.03)	−0.54 (−1.35, 0.28)	−1.36 (−3.40, 0.68)	−0.89 (−1.88, 0.10)	−0.42 (−1.08, 0.24)	−0.84 (−1.53, −0.16)
	First-onset schizophrenia	−0.55 (−1.35, −0.39)	−1.01 (−2.28, 0.26)	−2.19 (−3.81, −0.56)	−2.55 (−5.71, 0.61)	−1.52 (−3.04, 0.01)	−1.92 (−5.05, 1.21)
	Chronic schizophrenia	−0.87 (−1.35, −0.39)	−0.62 (−1.10, −0.15)	−1.22 (−2.08, −0.36)	−1.56 (−2.08, −1.03)	−0.50 (−0.97, −0.04)	−1.54 (−2.06, −1.02)
	Stable schizophrenia	−0.60 (−1.18, −0.02)	−1.10 (−1.80, −0.40)	−1.06 (−1.70, −0.41)	−0.80 (−1.24, −0.35)	−1.11 (−1.79, −0.43)	−0.87 (−1.46, −0.27)
Drug-naive	No	−0.67 (−0.91, −0.44)	−0.84 (−1.13, −0.55)	−1.56 (−2.03, −1.09)	−1.27 (−1.85, −0.69)	−0.93 (−1.25, −0.62)	−1.09 (−1.51, −0.68)
	Yes	−0.84 (−1.03, −0.64)	−1.15 (−1.39, −0.91)^*^	−1.13 (−1.79, −0.47)	−1.11 (−1.33, −0.88)	−0.83 (−1.91, −0.25)	−1.13 (−1.35, −0.90)
Setting	Outpatient & inpatient	−0.92 (−1.21, −0.63)	−1.22 (−1.58, −0.87)	−1.73 (−2.20, −1.25)	−1.07 (−1.26, −0.88)	−0.84 (−1.39, −0.28)	−0.90 (−1.08, −0.72)
	Inpatient	−0.56 (−0.80, −0.32)	−0.68 (−1.00, −0.35)	−0.98 (−1.28, −0.67)	−0.86 (−1.16, −0.55)	−1.12 (−1.63, −0.60)	−1.03 (−1.47, −0.60)
	Outpatient	−0.39 (−0.82, 0.04)	−0.42 (−0.68, −0.15)^*^	−3.52 (−7.20, 0.16)^*^	−3.67 (−7.87, 0.53)	−0.38 (−0.72, −0.03)^*^	−2.52 (−4.47, −0.56)
Facial emotion identification task	Validated in Chinese	−0.69 (−0.92, −0.45)	−0.98 (−1.26, −0.70)	−1.35 (−1.71, −0.98)	−1.02 (−1.20, −0.84)	−0.91 (−1.27, −0.55)	−0.99 (−1.19, −0.78)
	Not validated in Chinese	−0.71 (−1.03, −0.38)	−0.43 (−0.64, −0.23)^*^	−1.73 (−2.79, −0.67)	−2.11 (−4.53, 0.32)	−0.92 (−1.80, −0.05)	−1.72 (−3.63, 0.19)
Outcome measure	Correct rate	−0.74 (−1.03, −0.44)	−0.76 (−1.13, −0.38)	−1.45 (−2.11, −0.80)	−1.50 (−2.63, −0.27)	−0.99 (−1.48, −0.50)	−1.38 (−2.14, −0.63)
	Correct score	−0.65 (−0.91, −0.40)	−0.77 (−1.30, −0.64)	−1.46 (−1.89, −1.02)	−1.06 (−1.28, −0.84)	−0.84 (−1.31, −0.37)	−0.95 (−1.20, −0.69)
**Continuous moderator**							
Publication year		0.014 (−0.024, 0.052)	0.0010 (−0.0477, 0.0497)	0.092 (0.016, 0.169)^*^	0.041 (−0.047, 0.128)	0.064 (−0.131, 0.002)	0.020 (−0.043, 0.083)
Mean age of the patient sample		0.0053 (−0.0225, 0.0331)	0.0061 (−0.0401, 0.0279)	0.032 (−0.016, 0.080)	0.021 (−0.028, 0.070)	−0.025 (−0.065, 0.015)	0.0042 (−0.0322, 0.0405)
		**Happiness**	**Sadness**	**Fear**	**Disgust**	**Anger**	**Surprise**
Mean education years of the patient sample		0.052 (−0.098, 0.202)	0.12 (−0.05, 0.29)	−0.30 (−0.58, −0.03)^*^	−0.27 (−0.57, 0.03)	−0.029 (−0.228, 0.169)	−0.16 (−0.37, 0.06)
% of males in the patient sample		0.0089 (0.0013, 0.0166)^*^	0.0038 (−0.0077, 0.0153)	0.014 (−0.004, 0.0031)	0.0030 (−0.0181, 0.0242)	0.0052 (−0.0104, 0.0208)	0.0039 (−0.0110, 0.0188)
Mean PANSS positive subscale score of the patient sample		−0.060 (−0.107, −0.013)^*^	−0.057 (−0.126, 0.012)	0.072 (−0.057, 0.202)	0.11 (−0.03, 0.24)	0.0021 (−0.1114, 0.1157)	0.074 (−0.049, 0.197)
Mean PANSS negative subscale score of the patient sample		−0.025 (−0.075, 0.025)	−0.073 (−0.125, −0.021)^*^	0.064 (−0.057, 0.186)	0.12 (−0.03, 0.26)	−0.11 (−0.17, −0.04)^*^	0.020 (−0.088, 0.127)
Risk of bias score		−0.0033 (−0.1462, 0.1397)	0.0135 (−0.1884, 0.2154)	−0.17 (−0.44, 0.11)	−0.40 (−0.73, −0.06)^*^	0.19 (−0.06, 0.45)	−0.34 (−0.57, −0.10)^*^

* Statistically significant (P < 0.05) differences across subgroups or statistically significant (P < 0.05) coefficients. Because the number of studies examining contempt, calmness, and neutral emotions are lower than 10, sources of heterogeneity of these studies were not tested.

## Discussion

This study is a detailed quantitative systematic review of the FEI deficits with respect to nine emotions, which are potentially clinically relevant but have not been systematically examined in previously published studies. The main findings of this meta-analysis are the significantly lower FEI scores in Chinese PLwS than healthy controls in terms of the six basic emotions plus contempt, calm, and neutral, with the magnitudes of impairments being large for fear, disgust, surprise, anger, and sadness, being medium for happiness, and being small for contempt, calmness, and neutral. In the analyses of sources of heterogeneity, clinical factors, such as diagnostic criteria, drug-naïve status, clinical setting, and PANSS-N score, and methodology factors, such as FEI task and RoB score, were significant moderators of schizophrenia-control FEI performance differences.

Findings from empirical studies have shown that PLwS present more severe impairments in recognizing negative and neutral emotions, such as anger, fear, and calmness, while they do not present difficulties in recognizing positive emotions, such as happiness (1, 57, 58). Similarly, the meta-analysis found the greatest levels of impairments in identifying fear, disgust, anger, and sadness emotions in Chinese PLwS. However, the moderate level of impairment in identifying the emotion of happiness and the mild levels of impairment in identifying contempt, calmness, and neutral emotions seem to be not consistent with previous studies. These findings are partly attributed to the attentional biases to emotional scenes in PLwS; that is, compared to controls, PLwS showed increased attention to threatening scenes and paid less attention to happy scenes (59). In addition, the low levels of difficulty of the happiness items in the FEI tasks of prior studies might be the other possible explanation for this inconsistent finding because of the poor discriminant validity of the happiness items (53). As a supporting case in point, studies using validated FEI revealed a severe sadness-specific identification deficit in schizophrenia, but those using un-validated tasks only revealed a moderate sadness-specific identification deficit in our subgroup analyses (Table 3). Due to the limited number of included studies focusing on the FEI of neutral emotions, more empirical studies are warranted to ascertain the severity of impairments in recognizing neutral emotions in schizophrenia.

In line with earlier studies, the significant moderating roles of several clinical factors on the FEI abilities in schizophrenia were further confirmed (14, 16). Nonetheless, the findings are detailed enough, specific to the emotion in the FEI task. Although the effectiveness of antipsychotic treatment is limited for improving the facial affect processing deficits in schizophrenia, antipsychotic treatment still has a significant positive effect on FER deficits, and some second-generation antipsychotics can effectively relieve FER deficits in schizophrenia, particularly in terms of some negative emotions (60, 61). In keeping with this, more sadness-specific FEI impairments were found in drug-naïve than medicated PLwS in our subgroup analyses. Psychotic symptoms, both positive and negative symptoms, can negatively influence FER and processing (1, 15); therefore, our meta-regression analyses show significant negative correlations of the mean PANSS-P score with happiness-specific pooled SMDs, and the mean PANSS-N score with sadness-specific and anger-specific pooled SMDs. In general, inpatients have more psychotic symptoms than outpatients. In accordance with the negative associations between psychotic symptoms and pooled SMDs in the meta-regression analyses, the subgroup analyses found greater levels of sadness-, fear-, and anger-specific FEI difficulties in studies enrolling inpatients and both outpatients and inpatients than those enrolling outpatients only. Finally, one interesting finding from the subgroup analyses is the non-significant differences in pooled SMDs across clinical stages of schizophrenia, again confirming the trait characteristic of FEI impairment in PLwS.

The negative correlations between the RoB score and disgust- and surprise-specific FEI SMDs deserve to be emphasized because it suggests that the RoB of the included studies influences the magnitude estimates of FEI deficits in schizophrenia and that the magnitude of FEI impairments would be larger if more low-RoB studies were included in this meta-analysis.

This meta-analysis has several limitations. First, to minimize the own-race bias for FEI, the included studies were limited to those with participants of ethnic Chinese origin only. It is necessary to repeat our analyses in studies with participants from Western countries. The second significant limitation is the high RoB of the included studies since no included studies scored nine in the JBI checklist assessment. Third, the number of studies focusing on facial emotions other than the six basic emotions was small (*n* = 2–6), and our estimates of the magnitudes of FEI impairments in terms of these facial emotions might be unstable.

In summary, Chinese PLwS have FEI deficits in terms of all nine emotions of interest in this study, and the deficits are severe in terms of fear, disgust, surprise, anger, and sadness emotions, moderate in terms of happiness emotions, and mild in terms of contempt, calmness, and neutral emotions. Drug-naïve status, clinical setting, positive psychotic symptoms, and negative psychotic symptoms are potential moderators of the magnitudes of FEI deficits. It is necessary to consider these characteristics of FEI deficits and the clinical moderators of the FEI deficits when developing remediation strategies targeting FER deficits in schizophrenia.

## Data availability statement

The original contributions presented in the study are included in the article/Supplementary material, further inquiries can be directed to the corresponding author/s.

## Author contributions

Y-MX: acquisition and analysis of data for the study, drafting the paper, and interpretation of data for the study. Y-MX and FD: design and acquisition of data for the study. B-LZ: drafting the paper, revising the paper for important intellectual content, and interpretation of data for the study. All authors contributed to the article and approved the submitted version.
