# Supplementary material for: Facial emotion identification impairments in Chinese persons living with schizophrenia: A meta-analysis
Source: Front Psychiatry. 2022 Dec 20;13:1097350. doi: 10.3389/fpsyt.2022.1097350 (PMC9807786; doi:10.3389/fpsyt.2022.1097350)
Supplement: Supplementary file 1 [file Data_Sheet_1.docx]

**Figure legends:**

Supplementary figure 1. Forest plot of standardized mean differences in happiness-specific facial emotion identification scores between schizophrenia patients and healthy controls

Supplementary figure 2. Forest plot of standardized mean differences in sadness-specific facial emotion identification scores between schizophrenia patients and healthy controls

Supplementary figure 3. Forest plot of standardized mean differences in fear-specific facial emotion identification scores between schizophrenia patients and healthy controls

Supplementary figure 4. Forest plot of standardized mean differences in disgust-specific facial emotion identification scores between schizophrenia patients and healthy controls

Supplementary figure 5. Forest plot of standardized mean differences in anger-specific facial emotion identification scores between schizophrenia patients and healthy controls

Supplementary figure 6. Forest plot of standardized mean differences in surprise-specific facial emotion identification scores between schizophrenia patients and healthy controls

Supplementary figure 7. Forest plot of standardized mean differences in contempt-specific facial emotion identification scores between schizophrenia patients and healthy controls

Supplementary figure 8. Forest plot of standardized mean differences in calmness-specific facial emotion identification scores between schizophrenia patients and healthy controls

Supplementary figure 9. Forest plot of standardized mean differences in neutral-specific facial emotion identification scores between schizophrenia patients and healthy controls

Supplementary figure 10. Funnel plot of publication bias in standardized mean differences in happiness-specific facial emotion identification scores between schizophrenia patients and healthy controls

Supplementary figure 11. Funnel plot of publication bias in standardized mean differences in sadness-specific facial emotion identification scores between schizophrenia patients and healthy controls

Supplementary figure 12. Funnel plot of publication bias in standardized mean differences in fear-specific facial emotion identification scores between schizophrenia patients and healthy controls

Supplementary figure 13. Funnel plot of publication bias in standardized mean differences in disgust-specific facial emotion identification scores between schizophrenia patients and healthy controls

Supplementary figure 14. Funnel plot of publication bias in standardized mean differences in anger-specific facial emotion identification scores between schizophrenia patients and healthy controls

Supplementary figure 15. Funnel plot of publication bias in standardized mean differences in surprise-specific facial emotion identification scores between schizophrenia patients and healthy controls

Supplementary figure 1. Forest plot of standardized mean differences in happiness-specific facial emotion identification scores between schizophrenia patients and healthy controls





Supplementary figure 2. Forest plot of standardized mean differences in sadness-specific facial emotion identification scores between schizophrenia patients and healthy controls





Supplementary figure 3. Forest plot of standardized mean differences in fear-specific facial emotion identification scores between schizophrenia patients and healthy controls





Supplementary figure 4. Forest plot of standardized mean differences in disgust-specific facial emotion identification scores between schizophrenia patients and healthy controls





Supplementary figure 5. Forest plot of standardized mean differences in anger-specific facial emotion identification scores between schizophrenia patients and healthy controls





Supplementary figure 6. Forest plot of standardized mean differences in surprise-specific facial emotion identification scores between schizophrenia patients and healthy controls





Supplementary figure 7. Forest plot of standardized mean differences in contempt-specific facial emotion identification scores between schizophrenia patients and healthy controls





Supplementary figure 8. Forest plot of standardized mean differences in calmness-specific facial emotion identification scores between schizophrenia patients and healthy controls





Supplementary figure 9. Forest plot of standardized mean differences in neutral-specific facial emotion identification scores between schizophrenia patients and healthy controls





Supplementary figure 10. Funnel plot of publication bias in standardized mean differences in happiness-specific facial emotion identification scores between schizophrenia patients and healthy controls





Supplementary figure 11. Funnel plot of publication bias in standardized mean differences in sadness-specific facial emotion identification scores between schizophrenia patients and healthy controls





Supplementary figure 12. Funnel plot of publication bias in standardized mean differences in fear-specific facial emotion identification scores between schizophrenia patients and healthy controls





Supplementary figure 13. Funnel plot of publication bias in standardized mean differences in disgust-specific facial emotion identification scores between schizophrenia patients and healthy controls





Supplementary figure 14. Funnel plot of publication bias in standardized mean differences in anger-specific facial emotion identification scores between schizophrenia patients and healthy controls





Supplementary figure 15. Funnel plot of publication bias in standardized mean differences in surprise-specific facial emotion identification scores between schizophrenia patients and healthy controls
